# Incidence, cost and gender differences of oropharyngeal and noncervical anogenital cancers in South Korea

**DOI:** 10.1186/s12889-020-09161-y

**Published:** 2020-06-29

**Authors:** Inseon Choi, Donghwan Lee, Kyung-Bok Son, SeungJin Bae

**Affiliations:** 1 College of Pharmacy, Ewha Womans University, Seoul, South Korea; 2 Department of Statistics, Ewha Womans University, Seoul, South Korea

**Keywords:** Cost, Incidence, Burden of disease, Oropharyngeal cancer, Noncervical anogenital cancer

## Abstract

**Background:**

Human papillomavirus (HPV) is associated with a significant public health burden, yet few studies have been conducted in Asia, especially on noncervical cancers. We estimated the incidence and cost of oropharyngeal and noncervical anogenital (anal, vulvar, vaginal, penile) cancer in Korea.

**Methods:**

We conducted a retrospective cohort study using Korea’s National Health Insurance (NHI) claim database from 2013 to 2016. The main outcome measures were the number of respective cancer incidences during the study period and the annual costs per patient in the first year after diagnosis, which was adjusted by relevant variables based on the regression analysis.

**Results:**

During the study period, 8022 patients with these cancers were identified, and oropharyngeal cancer comprised 46% of them. The crude incidence rate for male oropharyngeal cancer was significantly higher than that of females (3.1 vs. 0.7 per 100,000 as of 2016, respectively). Additionally, the crude incidence of male oropharyngeal cancer increased from 2.7 in 2013 to 3.1 in 2016, whereas that of female and other cancers was stable during the study period. The mean annual incidence-based cost per patient in 2016 was highest for oropharyngeal cancers (21,870 USD), and it was significantly higher in males than in females based on then regression analysis (*p* < .001).

**Conclusions:**

Oropharyngeal cancer comprises the highest number of HPV-associated noncervical cancer incidences in Korea, and the incidence and cost of oropharyngeal cancer was significantly higher among males than females. More aggressive public health policy toward males may decrease gender gap of oropharyngeal cancer.

## Introduction

Cancer is a major cause of death in Korea, resulting in 82,155 deaths in 2018 [1]. Human papillomavirus (HPV) is attributable to 11.3% of cases and 6% of deaths of the infection-related cancers in Korea [2]. Currently, a 2-valent HPV vaccine (Cervarix™), 4-valent vaccine (Gardasil®), and 9-valent vaccine (Gardasil®9;) are available in Korea [3], and the National Immunization Program (NIP) has been implemented for 12-year-old girls with 2-valent HPV vaccine and 4-valent vaccine since June 2016 [4]. A previous Korean study using claim data suggested that the number of patients with diseases associated with HPV steadily increased between 2002 and 2015 [5]. In addition, in 2015, a total of 124.9 million USD was spent in Korea for healthcare costs for HPV-associated diseases [5]. Previous studies reported that the prevalence of cervical cancer per 100,000 decreased from 114.0 in 2007 to 90.8 in 2011. However, the prevalence of noncervical HPV-associated cancers, such as anal (2.7 to 3.5), vulvar (1.6 to 1.7), vaginal (1.4 to 1.5), and penile (0.7 to 0.9) cancers per 100,000, tended to increase from 2007 to 2011 [6].

Most HPV-associated cancer studies in Korea have estimated prevalence-based cost [5, 6], yet studies on incidence-based healthcare costs are scarce. Prevalence-based costs estimate the total cost of a disease during a particular year, including survivors and end-of-life patients [7–9]. Incidence-based costs estimate the cost of treatment for patients first diagnosed in a particular year [7–9]. Since a prevalence-based approach captures the medical expenditure that occurs during a specific period regardless of the timing of the disease incidence, its implication in estimating the burden of disease is limited [10]. However, incidence-based costs include only newly diagnosed patients, so it is possible to estimate health care costs after initial disease diagnosis [11]. Therefore, incidence-based cost estimation results are especially important for cancers, where most cost occur in the first year of the diagnosis [12–16]. The purpose of our study is to estimate the incidence rate and incidence-based healthcare cost of noncervical anogenital cancer and oropharyngeal cancer in Korea using nationally representative data.

## Materials and methods

### Database

We used the Health Insurance Review and Assessment (HIRA) database, which contains National Health Insurance (NHI) claims data in South Korea. The HIRA claims data cover almost 50 million patients and include utilization information regarding healthcare services reimbursed by the NHI (such as diagnosis code, cost of treatment, demographic characteristics, and prescription information) [17, 18]. The HIRA dataset includes 100% of Korean residents, with 97% covered by the NHI and 3% by the Medical Aid program, which is designed for the underprivileged [19].

Our study was approved by the Institutional Review Board of Ewha Womans University (IRB File No. 168–10).

### Study population

Patients with primary or secondary diagnosis of [6] oropharyngeal (including base of tongue, tonsil), anal, vulvar, vaginal, and/or penile cancer between January 2011 and December 2017 based on the International Classification of Diseases (ICD) code were identified from the NHI claim database. The ICD 10th codes for each cancer are as follows: oropharynx with base of tongue and tonsil (C01, C09, C10), anus (C21), vulva (C51), vagina (C52), and penis (C60).

**Fig. 1:**
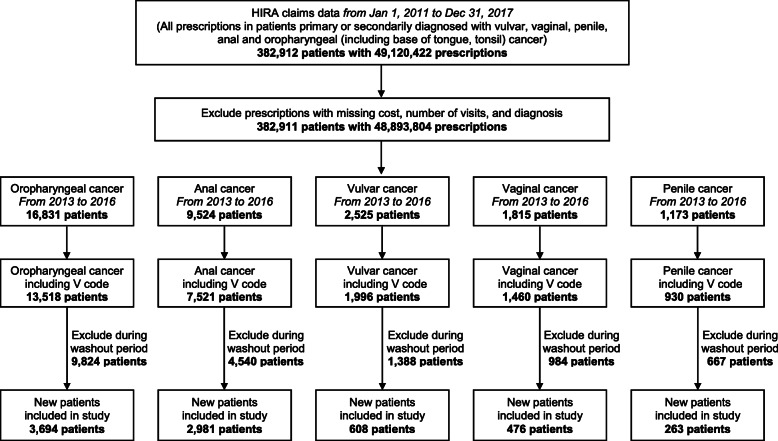
Flow chart of new patients included in the study through operational definition. Flow chart of extracting new patients from 2013 to 2016 through operational definition using HIRA claims data from patients diagnosed primary or secondary with vulvar, vaginal, penile, anal and oropharyngeal (including base of tongue, tonsil) cancer from Jan 1, 2011 to Dec 31, 2017

The washout period was defined as two years based on the clinicians’ advice that each patient could be defined as a new patient if there was no medical use for the same disease in the past two years based on the index date, which is the first diagnosed with corresponding cancers, from 2013 to 2016. Namely, to identify newly diagnosed patients, we excluded patients who had visited the medical institution with the same disease diagnosis during the two-year washout period (pre-index period). The follow-up period was defined as 1 year from the index date to estimate the annual cost per patient. The flow chart for the new patient included in the study through this operational definition is shown in Fig. 1. The Charlson comorbidity index (CCI) was used to adjust the comorbidities of patients [20].

### Outcomes

We estimated the number of newly diagnosed patients and incidence rates from 2013 until 2016 with 2011 and 2012 being used for the washout period, and analyzed the incidence-based medical cost per patient per year of oropharyngeal (including base of tongue, tonsil) and anogenital cancer (vulvar, vaginal, penile, anal cancer). We calculated the crude incidence rate per 100,000 people using the entire Korean population for the corresponding year as a denominator for new patients. The age-standardized incidence rate (ASR) per 100,000 people was calculated based on the World Health Organization (WHO)‘s 2000–2025 world standard population [21].

We calculated the direct medical cost from the Korean health care system perspective, which includes payer’s cost and patients’ cost sharing. Since Korean NHI has fee-for service system for most of the services except seven diseases areas (which are reimbursed by the Diagnosis Related Group), we estimated annual medical cost per patient by incorporating individual claim corresponds to each patient, including outpatient visits, laboratory tests, hospitalizations, and prescription medications [19] occurred within one-year following the index date. We estimated all costs by index year (2013–16). Because the diseases considered in this paper are rare, we included all patients in cost estimation. In order to summarize results less distorted by the outliers, we present the median of the cost estimates as well as the average.

All costs were adjusted by the medical care component of the Consumer Price Index in 2016 [22]. The cost was converted from Korean Won to US Dollar using the 2013–2016 average exchange rate (1USD = 1110 KRW) [23].

### Statistical analysis

Descriptive statistics were used to examine the overall distribution of the new patients for each cancer. To compare the medical cost differences between groups of independent variables, parametric tests (t-test, ANOVA test) and nonparametric tests (Wilcoxon test, Kruskal-Wallis test) were performed. Regression analysis was used to identify the association of medical costs with the independent variables. Independent variables included sex (female = 0, male = 1), age (under 65 or 65 = 0, 65 over = 1), surgical experience (inexperience = 0, experience = 1), health plan type (healthcare insurance = 0, Medicaid = 1), year and CCI. The surgical experience variable indicates whether or not the patient has undergone surgery during the follow-up period. Since disease severity information is not available at the NHI claim database, surgical experience was employed to adjust for the patients’ disease severity [24]. Because the distribution of medical costs was right-skewed, the log-transformation was considered [25, 26]. However, despite the conversion to the logarithm, Kolmogorov-Smirnov tests for checking normality failed for all cancer types (*p* < 0.05). Therefore, in addition to the linear regression model, generalized linear models (GLMs) were performed when the distribution of medical costs was assumed to be from gamma distribution. Gamma GLM fits a skewed distribution well and is frequently used for dealing with nonnormal data such as healthcare costs [27]. Using GLM, medical costs were analyzed by adjusting the effects of confounding factors such as age, surgical experience, health plan type, year, and CCI. Before adjusting for confounding factors, univariate analyses for each variable were also applied. After the analysis, regression models were compared based on the Akaike information criterion (AIC).

All data collection and statistical analysis were performed using SAS software (version 9.4, SAS Institute Inc., Cary, NC, USA) and RStudio (version 1.1.463, RStudio, Inc., USA). All analyses were conducted using *p* < 0.05 as the level of significance.

## Results

During the study period, 8022 new patients were identified, and the total number of patients with any of the five cancers increased from 1952 in 2013 to 2101 in 2016. Oropharyngeal cancer comprised 46% of them, followed by anal cancer (37%). The demographic characteristics of newly diagnosed patients for oropharyngeal cancer and anogenital cancers are presented in Table 1. For all five cancers, the mean age was greater than 60, and the CCI score was between 1 and 2. Most patients were covered by the NHI insurance (> 90%). There was a large gender gap in oropharyngeal cancer, with male patients comprising 81.2% (811 out of 999 patients) in 2016.

**Table 1:** Demographic characteristics of new patients for oropharyngeal and anogenital cancer, 2013–2016

Variable	2013	2014	2015	2016
**Oropharynx (C01, C09, C10)**^**a**^				
** N**	884	888	923	999
**Gender**, n (%)				
Male	698 (79.0)	730 (82.2)	737 (79.8)	811 (81.2)
Female	186 (21.0)	158 (17.8)	186 (20.2)	188 (18.8)
**Age**, mean (SD)	61.2 (12.2)	61.6 (12.5)	62.0 (12.5)	61.9 (12.5)
**CCI score**, mean (SD)	1.7 (1.3)	1.7 (1.2)	1.7 (1.2)	1.8 (1.2)
**Health plan type**, n (%)				
Healthcare insurance	824 (93.2)	839 (94.5)	868 (94.0)	927 (92.8)
Medicaid, etc. ^b^	60 (6.8)	49 (5.5)	55 (6.0)	72 (7.2)
**Anus (C21)**				
**N**	758	702	762	759
**Gender**, n (%)				
Male	388 (51.2)	404 (57.5)	425 (55.8)	376 (49.5)
Female	370 (48.8)	298 (42.5)	337 (44.2)	383 (50.5)
**Age**, mean (SD)	65.0 (12.9)	64.3 (13.5)	65.0 (13.8)	66.1 (13.3)
**CCI score**, mean (SD)	2.0 (1.1)	1.9 (1.2)	2.0 (1.2)	1.9 (1.2)
**Health plan type**, n (%)				
Healthcare insurance	704 (92.9)	652 (92.9)	688 (90.3)	688 (90.7)
Medicaid, etc. ^b^	54 (7.1)	50 (7.1)	74 (9.7)	71 (9.3)
**Vulva (C51)**				
**N**	147	156	156	149
**Age**, mean (SD)	62.0 (17.0)	58.7 (16.0)	64.3 (16.0)	64.0 (15.0)
**CCI score**, mean (SD)	1.2 (1.2)	1.2 (1.2)	1.1 (1.2)	1.3 (1.2)
**Health plan type**, n (%)				
Healthcare insurance	143 (97.3)	150 (96.2)	142 (91.0)	134 (90.0)
Medicaid, etc. ^b^	4 (2.7)	6 (3.8)	14 (9.0)	15 (10.0)
**Vagina (C52)**				
**N**	102	115	136	123
**Age**, mean (SD)	62.9 (13.5)	60.7 (14.3)	61.5 (14.3)	61.8 (14.6)
**CCI score**, mean (SD)	1.5 (1.2)	1.7 (1.2)	1.4 (1.2)	1.5 (1.3)
**Health plan type**, n (%)				
Healthcare insurance	96 (94.1)	105 (91.3)	127 (93.4)	112 (91.1)
Medicaid, etc. ^b^	6 (5.9)	10 (8.7)	9 (6.6)	11 (8.9)
**Penis (C60)**				
**N**	61	58	73	71
**Age**, mean (SD)	63.7 (16.0)	65.5 (15.4)	68.5 (12.6)	66.7 (14.8)
**CCI score**, mean (SD)	1.3 (1.2)	1.1 (1.2)	1.4 (1.1)	1.1 (1.1)
**Health plan type**, n (%)				
Healthcare insurance	58 (95.1)	56 (96.5)	70 (95.9)	64 (90.1)
Medicaid, etc. ^b^	3 (4.9)	2 (3.5)	3 (4.1)	7 (9.9)

*CCI* Charlson Comorbidity Index, *SD* standard deviation

^a^ Oropharyngeal cancer, including the base of tongue and tonsil

^b^ War veteran

Table 2 shows the crude and age standardized incidence rates of each cancers from 2012 to 2016. In general, although the crude incidence rates slightly increased during the study period, the trend disappeared when age standardized rates were used. During the study period, consistently higher number of male patients were observed in oropharyngeal cancer, with crude incidence rates per 100,000 for males vs. females being 3.1 vs. 0.7 in 2016, respectively (Table 2). Additionally, the crude incidence per 100,000 males increased from 2.7 in 2013 to 3.1 in 2016, but the incidence per 100,000 females did not change (0.7). A consistent pattern was seen in the age-standardized incidence rate, with the age-standardized incidence rates per 100,000 for males vs. females being 2.2 vs. 0.5 in 2016, respectively (Table 2). On the other hand, similar incidence rates for males and females were observed in anal cancer, and age-standardized incidence rates also showed consistent trend. For males, the crude incidence rate per 100,000 was highest for oropharyngeal cancer (3.1), followed by anal cancer (1.5) and penal cancer (0.3) in 2016. For females, the crude incidence rate per 100,000 was highest for anal cancer (1.5), followed by oropharyngeal cancer (0.7), vulvar cancer (0.6) and vaginal cancer (0.5) in 2016.

**Table 2:** Crude incidence rate (CR) and age-standardized incidence rate (ASR) of oropharyngeal and anogenital cancer

Cancer	Year	Male		Female	
		CR ^b^	ASR ^c^	CR ^b^	ASR ^c^
**Oropharynx** (C01,09,10) ^a^	2013	2.7	2.1	0.7	0.5
	2014	2.9	2.1	0.6	0.4
	2015	2.9	2.0	0.7	0.5
	2016	3.1	2.2	0.7	0.5
**Anus** (C21)	2013	1.5	1.2	1.5	0.9
	2014	1.6	1.2	1.2	0.7
	2015	1.7	1.3	1.3	0.8
	2016	1.5	1.0	1.5	0.9
**Vulva** (C51)	2013	–	–	0.6	0.4
	2014	–	–	0.6	0.4
	2015	–	–	0.6	0.4
	2016	–	–	0.6	0.4
**Vagina** (C52)	2013	–	–	0.4	0.3
	2014	–	–	0.4	0.3
	2015	–	–	0.5	0.5
	2016	–	–	0.5	0.3
**Penis** (C60)	2013	0.2	0.2	–	–
	2014	0.2	0.2	–	–
	2015	0.3	0.2	–	–
	2016	0.3	0.2	–	–

^a^ Oropharyngeal cancer, including the base of tongue and tonsil

^b^ Crude incidence rate is calculated per 100,000 persons

^c^ Age-standardized incidence rate is calculated per 100,000 persons; The age standardization method was a direct method and was adjusted to the World Standard Population of 2000–2025 by WHO

**Fig. 2:**
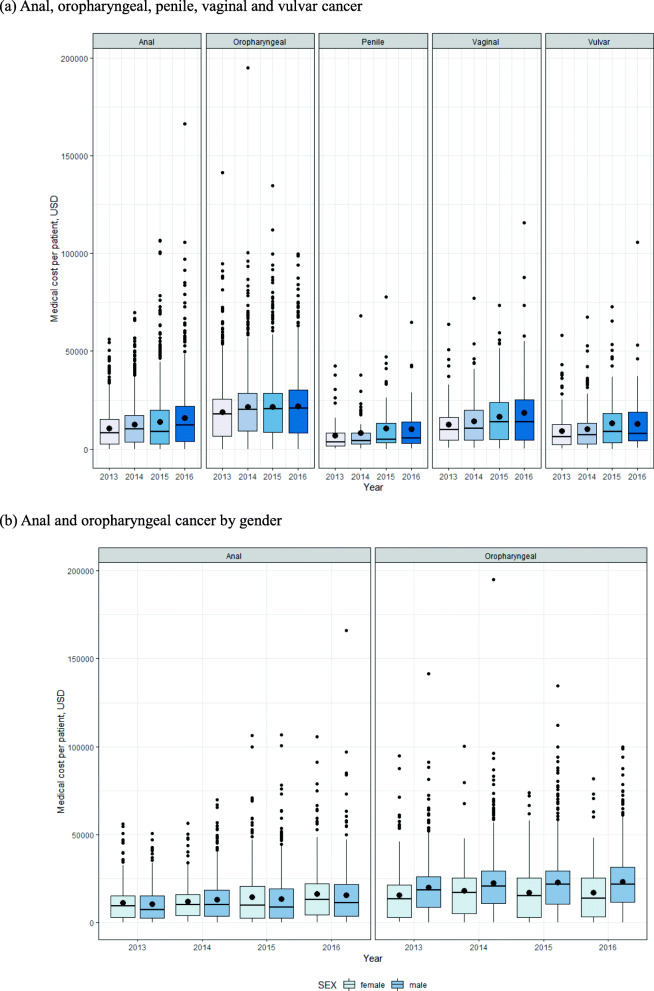
Incidence-based medical cost per patient, 2013–2016. **a** Anal, oropharyngeal, penile, vaginal and vulvar cancer. Incidence-based medical cost per patient in 2013–2016 of anal, oropharyngeal (including base of tongue, tonsil), penile, vaginal and vulvar cancer. All costs were adjusted by the medical care component of the Consumer Price Index in 2016. For the first year after diagnosis, the incidence-based cost per patient was highest in oropharyngeal cancer and lowest in penile cancer. In all five cancers, the incidence-based cost per patient increased between 2013 and 2016. **b** Anal and oropharyngeal cancer by gender. Differences in incidence-based medical cost per patient of anal cancer and oropharyngeal cancer according to gender. Oropharyngeal cancer demonstrated consistently higher results for males than for females during 2013–2016 for medical cost per patient. Anal cancer did not differ significantly between genders

The total incidence-based medical cost for oropharyngeal and noncervical anogenital cancer in 2013–2016 was 133,964,586 USD, bringing a significant economic burden to Korea, and greatly increased from 27,803,613 USD in 2013 to 38,864,428 USD in 2016. Table 3 and Fig. 2 show the incidence-based cost per patient for oropharyngeal cancer and anogenital cancer (anal, vulvar, vaginal, penile cancer). The incidence-based cost per patient was highest in oropharyngeal cancer and lowest in penile cancer. In all five cancers, the incidence-based cost per patient increased between 2013 and 2016. Among them, vaginal cancer showed the steepest increase from USD 12,515 in 2013 to USD 18,636 in 2016, followed by anal cancer, which increased from USD 10,662 in 2013 to USD 15,911 in 2016. The cost of oropharyngeal cancer was consistently higher for males than for females throughout the study period (Table 3, Fig. 2b). Specifically, the cost of oropharyngeal cancer per male patient was 23,041 USD whereas that per female being 16,819 USD in 2016. Accordingly, we analyzed cost per patient according to gender in oropharyngeal cancer through t-test and Wilcoxon test. The results demonstrated that the *p*-value of <.001 was significantly higher in male medical cost per patient than in females (Table 4).

**Table 3:** Incidence-based medical cost per patient of oropharyngeal and anogenital cancer in South Korea, 2013–2016

Cancer	2013		2014		2015		2016	
	Mean (USD)	Median (USD)	Mean (USD)	Median (USD)	Mean (USD)	Median (USD)	Mean (USD)	Median (USD)
**Oropharynx** (C01, 09, 10) ^a^	18,867	17,874	21,624	20,111	21,668	20,408	21,870	20,759
Male	19,719	18,445	22,418	20,635	22,828	21,492	23,041	21,606
Female	15,671	13,294	17,955	17,075	17,071	15,116	16,819	13,857
**Anus** (C21)	10,662	8195	12,630	10,105	13,737	9025	15,911	12,230
Male	10,331	7301	13,088	10,105	13,226	8645	15,419	11,042
Female	11,009	9379	12,008	10,115	14,382	9864	16,394	12,880
**Vulva** (C51)	9176	6188	10,261	7075	13,056	8869	12,957	8032
**Vagina** (C52)	12,515	9835	14,113	10,608	16,580	13,758	18,636	13,954
**Penis** (C60)	6852	3409	8137	4114	10,601	4961	10,097	5512

Unit: US dollar

Adjusted by the medical care component of the Consumer Price Index in 2016

^a^ Oropharyngeal cancer, including the base of tongue and tonsil

**Table 4:** Distribution of medical costs of independent variables

Variable	Oropharynx (C01, 09, 10) ^a^				Anus (C21)			
	Mean	p*	Median	p*	Mean	p*	Median	p*
**Sex**								
Female	16,837	<.001 ^c^	15,422	<.001 ^d^	13,528	0.295 ^c^	10,387	0.045 ^d^
Male	22,056		20,475		13,003		9196	
**Age**								
≤65	22,195	<.001 ^c^	20,644	<.001 ^d^	14,933	<.001 ^c^	10,978	<.001 ^d^
> 65	19,192		17,087		11,654		8710	
**Year**								
2013	18,867	0.001 ^e^	17,874	<.001 ^f^	10,662	<.001 ^e^	8195	<.001 ^f^
2014	21,624		20,111		12,630		10,105	
2015	21,668		20,408		13,737		9025	
2016	21,870		20,759		15,911		12,230	
**Surgery**								
Inexperience	12,243	<.001 ^c^	10,840	<.001 ^d^	7490	<.001 ^c^	3735	<.001 ^d^
Experience	24,011		22,210		16,216		12,744	
**Health plan type**								
Healthcare insurance	21,045	0.960 ^c^	19,762	0.348 ^d^	13,120	0.131 ^c^	9777	0.222 ^d^
Medicaid, etc. ^b^	20,990		18,106		14,655		10,399	
**CCI**								
0	22,718	<.001 ^e^	21,535	<.001 ^f^	15,040	<.001 ^e^	12,418	<.001 ^f^
1	23,993		22,091		15,101		12,154	
2	18,567		16,604		11,303		7729	
3	20,095		18,470		13,158		8781	

Unit: US dollar

*CCI* Charlson Comorbidity Index

* *p* < 0.05

^a^ Oropharyngeal cancer, including the base of tongue and tonsil

^b^ War veteran

^c^*p*-value estimated by t-test

^d^*p*-value estimated by Wilcoxon test

^e^*p*-value estimated by ANOVA test

^f^ p-value estimated by Kruskal-Wallis test

Regression analysis was performed on oropharyngeal cancer and anal cancer, which can occur in both females and males. Table 4 shows the distribution of medical costs for each category of independent variable, and the regression analysis results are shown in Table 5. In multivariate regression analysis, the cost of oropharyngeal cancer was significantly higher in males than in females after adjustment for confounding factors such as age, year, surgery, health plan type, and CCI (*p* < .001). Both the linear regression model and generalized linear model showed similar results. Also, the linear regression model showed better suitability as a result of comparing the goodness-of-fit of two multivariate models using the AIC.

**Table 5:** Linear regression model and generalized linear model for incidence-based medical cost

Model	Variable	Univariate				Multivariate			
		Anal		Oropharynx ^a^		Anal		Oropharynx ^a^	
		β	p*	β	p*	β	p*	β	p*
Linear Regression Model	**Sex** (ref = female)	−0.104	0.024	0.483	<.001	−0.150	0.004	0.422	<.001
	**Age** (ref = ≤65)	−0.180	<.001	−0.255	<.001	−0.136	0.001	−0.185	<.001
	**Year**	0.102	<.001	0.053	0.002	0.101	<.001	0.046	0.003
	**Surgery** (ref = inexperience)	1.040	<.001	1.001	<.001	1.037	<.001	0.956	<.001
	**Health plan type** (ref = healthcare insurance)	0.113	0.174	−0.118	0.131	0.115	0.131	−0.121	0.091
	**CCI**	−0.065	0.001	−0.079	<.001	0.003	0.987	−0.045	0.002
	AIC					95,597		12,2571	
Generalized Linear Model	**Sex** (ref = female)	−0.040	0.286	0.270	<.001	−0.088	0.012	0.249	<.001
	**Age** (ref = ≤65)	−0.248	<.001	−0.145	<.001	−0.120	<.001	−0.118	<.001
	**Year**	0.129	<.001	0.043	0.001	0.130	<.001	0.038	0.002
	**Surgery** (ref = inexperience)	0.773	<.001	0.674	<.001	0.759	<.001	0.657	<.001
	**Health plan type** (ref = healthcare insurance)	0.111	0.098	−0.003	0.965	0.129	0.040	0.013	0.823
	**CCI**	−0.050	0.001	−0.052	<.001	−0.009	0.534	−0.034	0.003
	AIC					103,891		132,144	

*AIC* Akaike Information Criterion, *CCI*, Charlson Comorbidity Index

* p < 0.05

^a^ Oropharyngeal cancer, including the base of tongue and tonsil

## Discussion

Our study estimated the incidence and cost of oropharyngeal cancer (including base of tongue, tonsil) and anogenital cancer (anal, vulvar, vaginal, penile cancer) in 2013–2016 using nationally representative sample in Korea. During the study period, 8022 new patients were identified, and the total number of patients with five cancers increased from 1952 in 2013 to 2101 in 2016. The crude incidence rate for male oropharyngeal cancer was the highest among the five cancers, and oropharyngeal cancer showed a significant gender gap, with males vs. females being 3.1 vs. 0.7 per 100,000 as of 2016, respectively. A similar pattern was seen in the age-standardized incidence rate, with males vs. females being 2.2 vs. 0.5 per 100,000 as of 2016, respectively. A previous Korean study also showed that the number of male patients was higher than that of female patients, which is similar to our study [5]. Additionally, the crude incidence of oropharyngeal cancer per 100,000 males increased from 2.7 in 2013 to 3.1 in 2016, whereas that of female and other cancers was stable during the study period. The incidence rate of oropharyngeal cancer associated with HPV is increasing in Asian countries such as Singapore, Taiwan and developed countries such as Northern Europe, Australia, and the United States, and the incidence rate is 2–3 times higher in males than in females [28–31], which is consistent with our study.

Vaginal cancer and anal cancer demonstrated the steepest increase in medical costs during 2013–2016 (12,515 USD in 2013 to 18,636 USD in 2016 (49%); 10,662 USD in 2013 to 15,911 USD in 2016 (49%), respectively). The cost of oropharyngeal cancer was the highest among the five cancers, which is consistent with a previous study [32]. Oropharyngeal cancer is anatomically complex and difficult to operate compared to other cancers, and reconstruction may be added to restore function [33]. Therefore, the cost of surgery is expected to be higher than that of other cancers. Additionally, Targeted anticancer drug cetuximab has been reimbursed since 2014, which is in line with a sudden increase in expenditure in 2014, whereas only existing chemotherapy is reimbursed for the rest of the other cancers [34]. Moreover, because the head and neck are the organs that are used for speaking and swallowing food, it is closely related with patients’ quality of life, and there is a risk of having a disability even after treatment such as neck resection [35]. Therefore, aggressive prevention of oropharyngeal cancer should be considered to improve health outcomes as well as reduce financial burden. Regression analysis was conducted in oropharyngeal cancer and anal cancer to estimate gender-specific annual cost, since those two cancers can occur in both genders. The cost of oropharyngeal cancer for males was significantly higher than that of females based on the regression analysis (*p* < .001), both in univariate and multivariate analysis. Higher cost for males was also found in previous studies [5, 36]. An American study found that female head and neck cancer patients did not receive active treatment compared to male counterpart [36]. In addition, a Korean study analyzed the medical costs of HPV-related diseases in 2015, and reported that the proportion of males using radiation therapy and operation was higher than that of females [5]. Also, given that smoking is closely related with oropharyngeal cancer and smoking prevalence for males is higher than that of females, smoking might be related our finding [37–39]. Thus, under-treatment of female patients, along with high smoking prevalence of males, might be attributable to the gender difference. Future study which includes smoking variable is needed.

It is not surprising that annual costs were significantly higher as years pass and for the patient with higher disease severity (defined based on surgery experience). Interestingly, our analysis showed that patients under 65 and a lower CCI had significantly higher cost based on the multivariate analysis, using both the GLM and OLS models. Since we defined cancer patients based on primary or secondary diagnosis only, patients who are defined to have respective cancers based on the tertiary or beyond diagnoses codes are not included in our analysis. Since those patients are likely to be older and have higher CCI scores, our study might underestimate the cost of patients with higher CCI or older age; thus, our study should be interpreted with caution.

A previous prevalence-based cost-of-illness study estimated the health care costs of HPV-associated diseases in Korea using claims data in 2015 and suggested that the number of patients for anal, vulvar, vaginal and penile cancer was 2071, 588, 383, and less than 300, respectively [5]. Since we estimated the new patients of each cancer, our estimates are lower than the prevalence of each cancer, yet the trend observed in their study is consistent with what we have estimated in the number of incidences of each cancer. Since our definition of oropharyngeal cancer (C01, C09, C10) is broader than that of the previous study (C10), caution should be taken when making comparison between the two studies. The prevalence-based healthcare costs per patient in a previous study were estimated at 4096 USD for oropharyngeal, 3737 USD for vaginal, 3370 USD for vulvar, 2807 USD for anal and 2169 USD for penile in 2015. The cost of oropharyngeal cancer was the highest, and the cost of penile cancer was the lowest, which is consistent with our study. The prevalence-based cost estimated in a previous study and the incidence-based cost estimated in our study are nearly five times different. This difference appears to be due to the high initial treatment cost of cancer after the first diagnosis [12, 13, 15, 16]. As such, the cost of incidence and prevalence-based cost are quite different and should not be confused.

The HPV associated annual cost per patient in 2016 was estimated to be 14,536 USD for vaginal cancer, 14,002 USD for anal cancer, 6736 USD for oropharyngeal cancer, 5049 USD for penile cancer and 3226 USD for vulvar cancer, respectively, after accounting for the HPV attributable fraction [28]. Overseas countries estimated the incidence rate and medical cost of HPV-associated noncervical cancers. A Danish study estimated the crude annual incidence rate of HPV-associated anogenital cancers (anal, penile, vaginal, vulvar cancer) in Denmark in 2004–2007 and suggested that the crude incidence rates for anal, penile, vaginal and vulvar cancer were 1.9, 1.7, 0.9 and 3.6 per 100,000 persons, respectively [15], which was higher than what we observed in our study (1.5, 0.3, 0.5, 0.6 for each year 2016, respectively) [15]. According to another Danish study, the incidence of anal cancer in Denmark has been steadily increasing, presumably due to patterns of change in sexual behavior [40]. The sex culture of Korea has changed rapidly in recent years, and the trend of an open sex culture is spreading due to the rapid westernization of social culture, which explains our increasing trend [41]. Therefore, appropriate prevention should be considered to prevent the increase in the incidence rate of HPV-associated disease in Korea. The incidence of oropharyngeal disease was estimated in Singapore, and the crude incidence per 100,000 persons of oropharyngeal squamous cell carcinoma was 2.66 for males and 0.72 for females in 2008–2012, which translates into 2.44 for males and 0.44 for females for the ASR per 100,000 [30]. Given that our ASR per 100,000 were 2.1 for males and 0.4 for females, our finding is a little bit lower than that of the Singaporean study. The Singaporean study suggested that Singapore has a potential burden of male oropharyngeal cancer and that changing current HPV precautions that focus on cervical cancer may also help prevent male oropharyngeal cancer. The American study calculated incidence-based medical costs for HPV-associated disease in 2004–2007 and reported that the annual direct medical costs per case was highest for the oropharyngeal cancer (43,200 USD) and lowest for the penile cancer being the lowest (19,800 USD) [42], which is consistent with our study. In terms of the absolute value, our values are relatively low in all disease areas. The United States mainly employs private health insurance and the charges for service are relatively high [43, 44] whereas Korea has national health insurance and the fee for medical services in Korea is around 60–80% of the original price [45]. Therefore, it is not surprising that our medical cost estimates in terms of the absolute value are relatively low compared with those of the American study.

Our study has some limitations. The cost of disease may be underestimated because non-medical costs, such as transportation costs, caregiver costs, and productivity losses are excluded. Second, we defined patients based on the primary or secondary diagnosis, so disease recorded after secondary diagnosis was excluded. Therefore, the result of estimating medical costs may vary according to such operational definition. Although large data sets are used, the CCI used in the regression analysis may depend on these operational definitions. Third, The HIRA database does not provide information on smoking status, tumor stage, or HPV status. Considering that smoking can exacerbate the progression of cancer, it is a limitation of our study. We included the surgery variable in the regression model to adjust for the disease severity, and assumed that experiencing surgery indicated higher cancer severity, which is a clear limitation. In addition, we could not tell whether the cancer patients are having primary cancer or metastasis, based on the NHI claim database. Since patients with metastatic cancer receive different treatment methods than those diagnosed with non-metastatic cancer, the cost can be different. Last, future studies reporting recurrence costs are needed.

However, our study has the following strengths. HIRA claim database contains comprehensive health care utilization information for almost 50 million Koreans, which provide an optimal environment to conduct a population-based study for rare diseases. In addition, 99% of HIRA claims data are claimed through electronic data interchange (EDI) [18]. Therefore, it is expected that almost all patients who used medical services for oropharyngeal and anogenital cancer (anal, vulvar, vaginal, penile cancer) during 2013–2016 would be included [46].

To our knowledge, our study is the first to estimate the incidence-based medical cost for oropharyngeal (including base of tongue, tonsil) and anogenital cancer (anal, vulvar, vaginal, penile cancer) in Asia. It is also the first to show that the incidence-based medical costs of oropharyngeal and anal, vulvar, vaginal, and penile cancers are increasing in Korea. The incidence-based medical cost estimation results in our study are particularly useful when considering disease prevention and can be useful as a basis for providing estimates of the potential costs [47, 48].

## Conclusion

The incidence-based cost per patient of oropharyngeal cancer (including the base of tongue, tonsil) and anogenital cancer (anal, vulvar, vaginal, penile cancer) increased from 2013 to 2016. Specifically, the incidence of oropharyngeal cancer was significantly higher in men, and costs were significantly higher in men than in women. More aggressive public health policy toward males may decrease the gender gap of oropharyngeal cancer.

## Data Availability

The data that support the findings of this study are available from [Health Insurance Review and Assessment Service] but restrictions apply to the availability of these data, which were used under license for the current study, and so are not publicly available. Data are however available from the authors upon reasonable request and with permission of [Health Insurance Review and Assessment Service].
